# Biomechanical behaviour of a novel bone cement screw in the minimally invasive treatment of Kummell's disease: a finite element study

**DOI:** 10.1186/s12891-023-07090-6

**Published:** 2023-12-14

**Authors:** Hanpeng Xu, Qing Feng, Xiang Ma, Jie Lan, Jingtao Ji, Zepei Zhang, Jun Miao

**Affiliations:** 1 Tianjin Hospital, Tianjin University, Tianjin, China; 2 Department of Orthopaedics, Union Hospital, Tongji Medical College, Huazhong University of Science and Technology, Wuhan, China

**Keywords:** Kummell’s disease, Osteoporosis, Bone cement, Spine, Finite element analysis

## Abstract

**Objective:**

To investigate and evaluate the biomechanical behaviour of a novel bone cement screw in the minimally invasive treatment of Kummell's disease (KD) by finite element (FE) analysis.

**Methods:**

A validated finite element model of healthy adult thoracolumbar vertebrae T12-L2 was given the osteoporotic material properties and the part of the middle bone tissue of the L1 vertebral body was removed to make it wedge-shaped. Based on these, FE model of KD was established. The FE model of KD was repaired and treated with three options: pure percutaneous vertebroplasty (Model A), novel unilateral cement screw placement (Model B), novel bilateral cement screw placement (Model C). Range of motion (ROM), maximum Von-Mises stress of T12 inferior endplate and bone cement, relative displacement of bone cement, and stress distribution of bone cement screws of three postoperative models and intact model in flexion and extension, as well as lateral bending and rotation were analyzed and compared.

**Results:**

The relative displacements of bone cement of Model B and C were similar in all actions studied, and both were smaller than that of Model A. The minimum value of relative displacement of bone cement is 0.0733 mm in the right axial rotation of Model B. The maximum Von-Mises stress in T12 lower endplate and bone cement was in Model C. The maximum Von-Mises stress of bone cement screws in Model C was less than that in Model B, and it was the most substantial in right axial rotation, which is 34%. There was no substantial difference in ROM of the three models.

**Conclusion:**

The novel bone cement screw can effectively reduce the relative displacement of bone cement by improving the stability of local cement. Among them, novel unilateral cement screw placement can obtain better fixation effect, and the impact on the biomechanical environment of vertebral body is less than that of novel bilateral cement screw placement, which provides a reference for minimally invasive treatment of KD in clinical practice.

## Introduction

Part of patients with osteoporotic vertebral compression fractures who do not undergo effective conservative methods or minimally invasive surgical treatment may develop progressive kyphosis and be accompanied by symptoms of spinal cord compression, known as Kummell's disease (KD). The number of these patients has been increasing in recent years. Some scholars [1, 2] believe that Kummell's disease is a complication or end-stage clinical manifestation of vertebral compression fractures, and once the onset will have a serious impact on the patient's life and quality of life, so more aggressive surgical treatments are required.

Patients with stage I, II, and III of Kummell's disease without neurological symptoms can be treated with minimally invasive surgery for percutaneous vertebroplasty (PVP) or balloon kyphoplasty (PKP) [3]. Although the treatment of PVP and PKP can stabilize the vertebral body and relieve pain in a short time, it is easy to cause loosening of bone cement during activity, and in severe cases, displacement and rupture may occur [4]. Mainly because the bone cement is mostly filled in the vertebral fracture, an envelope has been formed in the fissure, and the surrounding vertebral bone trabeculae are not closely combined, so that the bone cement cannot be firmly anchored to the bone tissue.

Anchoring of bone cement with internal fixation is a new way to treat KD. Pedicle screws have been widely used in clinical practice, and their safety and firmness have been confirmed [5]. The implantation technique of pedicle screw is combined with PVP. The anterior end of the screw is implanted into the bone cement and fused with the bone cement to stabilize the bone cement mass and prevent the bone cement from loosening and displacement. Existing pedicle bone cement screws cannot be implanted percutaneously or minimally, and the tail of the nail has a larger U-shape which can cause great disturbance to the surrounding soft tissue and can cause some symptoms, such as pain, foreign body sensation. To solve these clinical dilemmas, we designed a bone cement pedicle screw for the treatment of Kummell's disease.

**Fig. 1:**
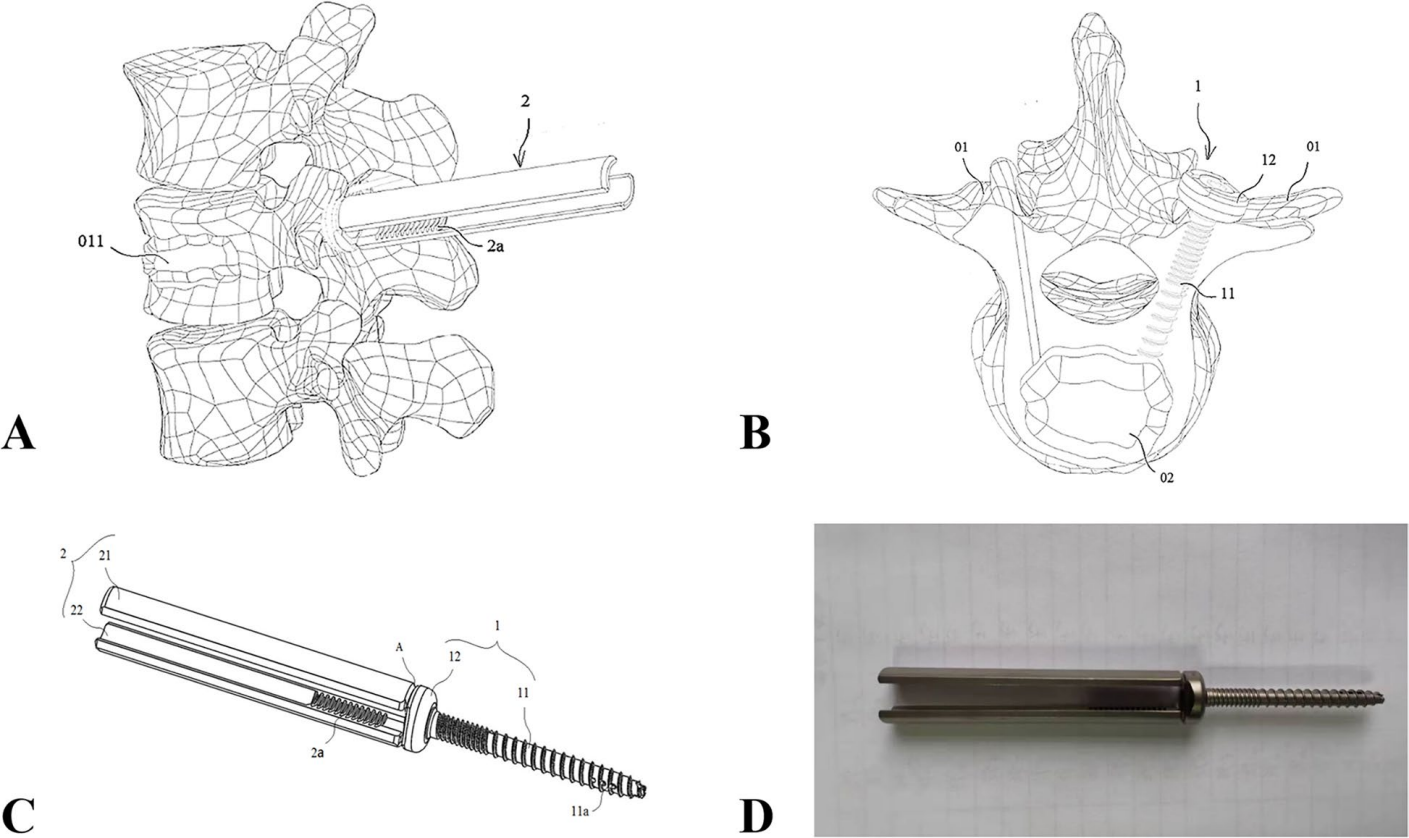
Novel minimally invasive pedicle cement screw **A**) Side view of the nailing process **B**) Vertical view after nail placement **C**) Structure pattern diagram **D**) finished product(01- pedicle; 011-Kummell's disease cavity; 02-bone cement mass; 1- screw body; 11-threaded connection part; 11a- Overflow hole; 12-head; 2-connecting arm; 21-first connecting arm; 22-s connecting arm; 2a- thread; A- Broken slot)

The novel bone cement screw (Fig. 1) has the following features: (1) the bone cement pedicle screw is a hollow screw, which is convenient for the insertion and injection of the guide needle and bone cement; (2) this screw has a long tail, which is conducive to percutaneous minimally invasive operation; (3) the nail tail is unidirectional, which can accurately connect the bone cement injector along the long tail; (4) this screw does not need to fix the connecting rod, and the easily fold part is at the root of the nail tail, so as to prevent the excessive nail tail left in the body to stimulate the soft tissue of patients and cause corresponding symptoms; (5) the thread part of the pedicle screw of this bone cement is a double-threaded design to enhance the screw holding force and prevent the failure of the internal fixation in patients with osteoporosis; (6) the distal end of the thread is designed with some bone cement leakage side holes where the bone cement injected into the screw under the X-ray will flow out of the screw side holes and fuse with the bone cement injected by PVP or PKP.

The purpose of this study is to investigate the anchoring effect of the novel minimally invasive bone cement screw on bone cement and the biomechanical performance of unilateral and bilateral anchoring groups in daily activities after treatment of Kummell's disease. Finite element (FE) technique is a technique which makes use of finite element to approach the real world infinitely. It is widely used in orthopedic research. It can accurately and intuitively simulate the forces on the implant under specific conditions and the biomechanical data of the spine related structures. In this study, the FE was used to investigate the biomechanical properties of the novel minimally invasive bone cement screw, in order to provide a reference for the selection of implants in Kummell's disease.

## Materials and methods

### Novel minimally invasive pedicle bone cement screw

The novel pedicle bone cement screw comprises an axially connected screw body and a connecting arm for connecting with surgical instruments (Fig. 1). The screw body comprises a threaded joint part and a head. The front end of the threaded joint part and the lateral side are provided with some overflow holes. The connecting arm and the head are connected in a detachable way.

After surgery, the connecting arm of the pedicle bone cement screw is disassembled, and the screw body remain in the patient. The threaded part is strongly connected with the bone cement mass, and ensures that the bone cement mass and the pedicle bone cement screw can’t shift by the force between the head and the posterior edge of the pedicle. On the other hand, since the connecting arm is disassembled, it can reduce irritation to the surrounding soft tissue, so as to avoid pain, foreign body sensation and other symptoms to the patient. During the operation, only about 1.5 cm incision is needed without the need to peel off muscle tissue, and surgeons are able to perform minimally invasive operations.

### FE model of healthy adult thoracolumbar vertebrae (T12-L2)

In this study, a FE model of thoracolumbar vertebrae (T12-L2) was build based on a healthy 26-year-old male volunteer with no history of spinal trauma or surgery. The volunteer underwent anteroposterior and lateral radiographs of the thoracolumbar vertebrae and showed no obvious degeneration. 3-dimensional (3D) CT data of the spine was scanned by the Imaging Department of Tianjin Hospital. Images of three vertebrae and two intervertebral discs between T12 and L2 were obtained using a 64-slice spiral computed tomography scanner (Siemens, Erlangen, Germany) with 0.625 mm interlayer spacing. DICOM images were imported into Mimics 20.0(Materialise Inc., Leuven, Belgium) to create a 3D vertebral body surface model T12—L2 [6]. 3-Matic 12.0 software (Materialise Inc.) was used to construct facet joints and annulus fibrosus stroma and nucleus pulposus. Materialize with the Geomagic Studio 2015 (Geomagic Inc., USA) software [7]. Hypermesh2017(Altair Engineering, Troy, Michigan, USA) was used for the reticulated construction of bone, disc, and ligament structures. Abaqus2020(Abaqus Inc., USA) was applied to add material attributes to the model, set analysis steps, load loads and simulation analysis [8].

**Fig. 2:**
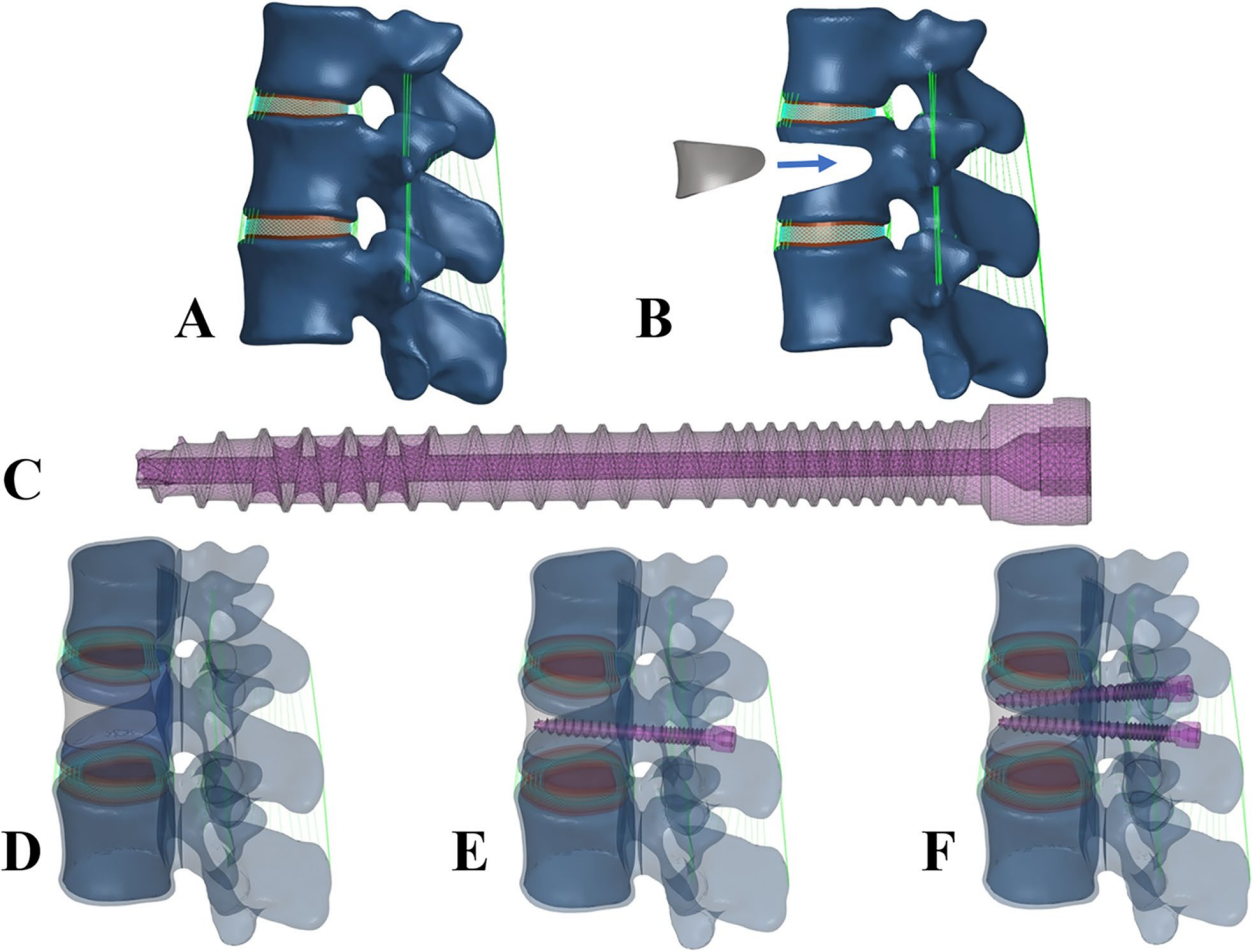
**A**) FE model of healthy adult thoracolumbar vertebrae (T12-L2); **B**) FE model of Kummell's disease after bone cement filling; **C**) FE model of a novel minimally invasive bone cement screw; **D**) FE model of pure percutaneous vertebroplasty (PVP group); **E**) FE model of the unilateral percutaneous novel minimally invasive pedicle bone cement screw anchoring group (unilateral anchoring group); F) FE model of bilateral percutaneous novel minimally invasive pedicle bone cement screw anchoring group (bilateral anchoring group)

The FE model of T12-L2 vertebrae of the subject is shown in Fig. 2. After mesh convergence verification, the balance between accuracy and calculation cost was achieved [9], and finally the mesh size was determined to be 1.0 mm, and the total number of elements in the complete T12-L2 model was 1,054,255. Hexagonal mesh was used for intervertebral disc and tetrahedral mesh was used for vertebral body [10]. The thickness of cortical bone, articular cartilage and cartilage endplate were 1 mm, 0.2 mm and 0.5 mm, respectively [9, 11]. The intervertebral disc was divided into the nucleus pulposus and the annulus fibrosus. The nucleus pulposus accounted for between 30 and 40% of the intervertebral volume [12]. The annulus fibrosus consists of annulus matrix and collagen fibers, which are divided into 5 layers at 25–45° from the horizontal surface. Each segment simulated seven ligaments, including the anterior longitudinal ligament (ALL), posterior longitudinal ligament (PLL), ligaments flavum (LF), articular capsular ligament (CL), intertransverse ligament (ITL), interspinous ligament (ISL), and supraspinous ligament (SSL) [13]. The friction coefficient of facet joint was set at 0.1 [10]. All ligaments were modelled using the Truss unit and are affected only by tensile loads [14]. The material properties of each section are derived from the published literature [10, 13, 15] (Table 1).

**Table 1:** Material properties defined in the T12-L2 FE model (ALL, anterior longitudinal ligament; PLL, posterior longitudinal ligament; LF, ligamentum flavum; ISL, interspinous ligament; supraspinous ligament, SSL; intertransverse ligament, ITL; CL, cystic ligament)

Component	Young’s modulus (MPa)	Poisson’s ratio	Cross-sectional area (mm^2^)
Bony structures			
Cortical bone	Osteoporotic:8040 (67% of normal) Normal:12,000	0.3	-
Cancellous bone	Osteoporotic:34 (34% of normal) Normal:100	0.2	-
Posterior structure	Osteoporotic:2345(67% of normal) Normal:3500	0.3	-
Endplate	Osteoporotic:670(67% of normal) Normal:1000	0.4	-
Facet cartilage	10	0.4	-
Intervertebral disc			
Annulus fibre	450	0.45	0.15
Annulus ground	4.2	0.45	-
Nucleus pulposus	1	0.49	-
Ligaments			
ALL	12.8	-	63.7
PLL	7	-	20
LF	3	-	40
ISL	6	-	40
SSL	6.6	-	30
ITL	7	-	1.8
CL	4	-	30
Implants			
Ti	110,000	0.3	-
PMMA	3500	0.3	-

The lower surface of L2 vertebral body was fixed and a 7.5Nm torque load was applied to the upper surface of T12. The range of motion (ROM) in each direction was measured and compared with previous studies published by Lu et al. [16] and W.Schmoelz et al. [17].

### FE model of osteoporosis and Kummell's disease

**Fig. 3:**
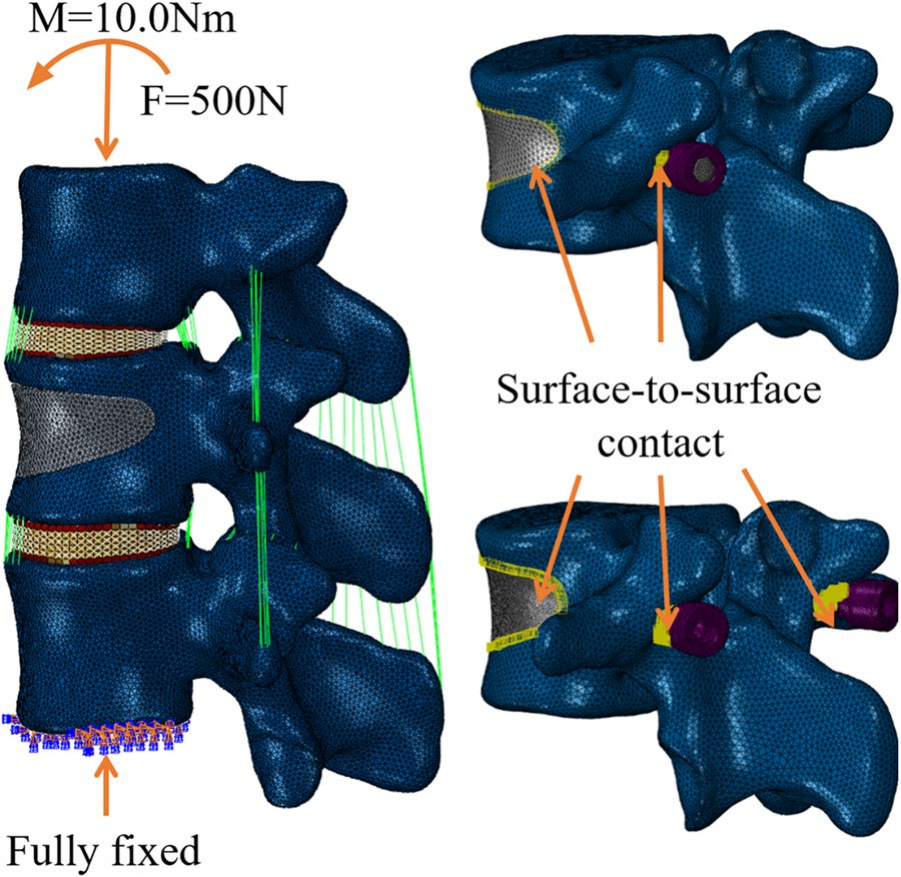
Loading mode and interaction type of finite element model

The validated FE model of healthy adults was modified. The model was endowed with material properties of osteoporosis [18, 19]. The wedge was removed from some of the bone tissue in the middle of the L1 vertebra to simulate the loss of bone tissue after a compression fracture (Fig. 2). The defect was filled with bone cement to simulate the ideal condition of the reconstructed vertebra after surgery. The novel minimally invasive bone cement screw performed ‘Boolean operation’ with bone cement and L1 vertebra. The interaction between them was set as surface-to-surface contact. The friction coefficient between bone cement, screw and vertebral body was set at 0.3 [15] (Fig. 3). The FE model of KD was repaired and treated with three options: pure percutaneous vertebroplasty (Model A), novel unilateral cement screw placement (Model B), novel bilateral cement screw placement (Model C), as shown in Fig. 2.

In the three models, the lower surface of L2 was fixed and the upper surface of T12 was applied with 500N of preload and 10.0Nm of torque to simulate the flexion, extension, lateral bending and axial rotation movements of the spine during daily activities. The ROM, the stress distribution of endplate, bone cement and screw, and the relative displacement of bone cement were recorded and compared among the three postoperative models.

## Results

### Validation of the healthy adult T12-L2 FE model

**Fig. 4:**
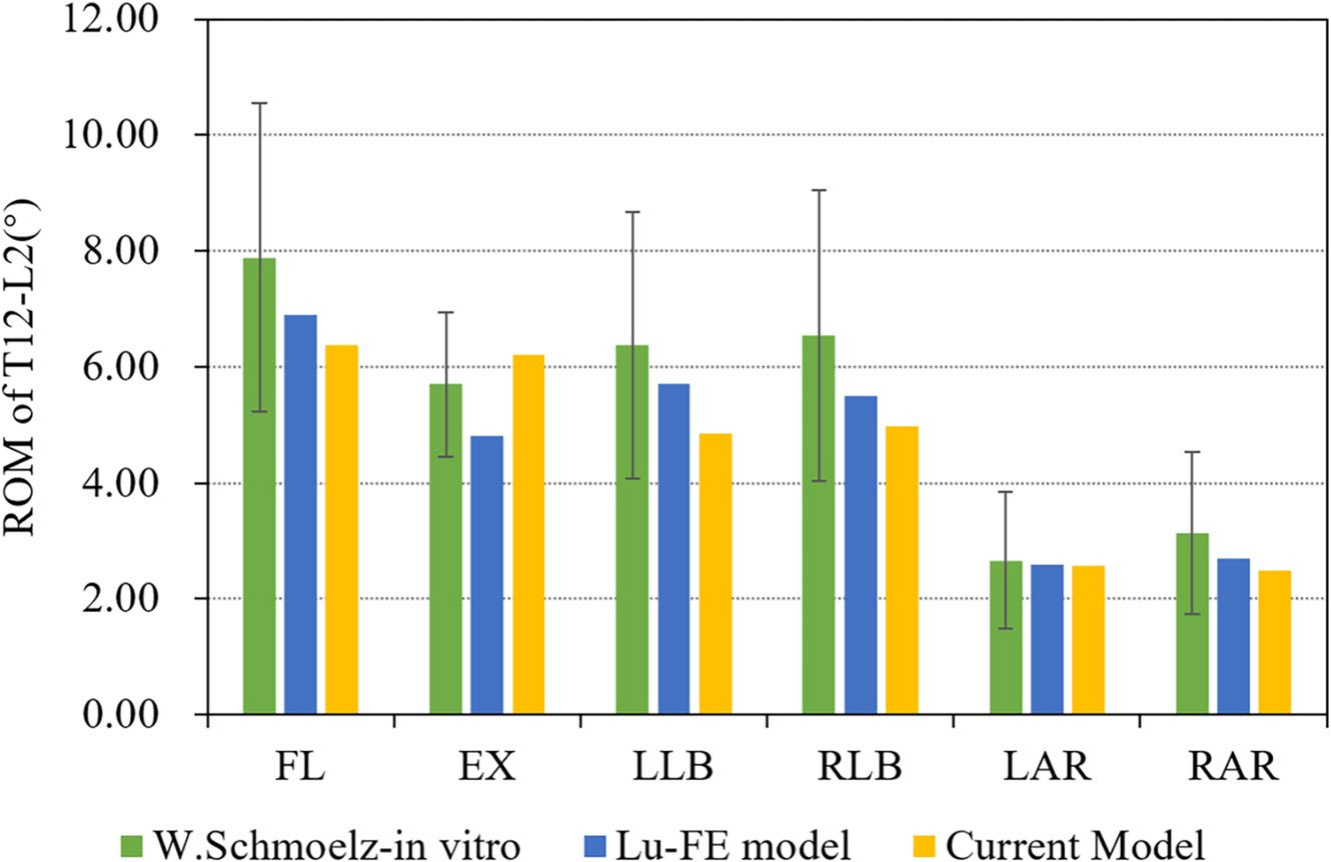
Comparison of ROM obtained from the current Model And reported data of in vitro experiments and FE studies (FL: flexion, EX: extension, LLB: left lateral bending, RLB: right lateral bending, LAR: left axial rotation, RAR: right axial rotation)

Compared with previous in vitro experiments and FE studies, ROM obtained from our model were in the same range of those reported by W.Schmoelz et al. [17]. In all activities, ROM of flexion was maximum and ROM of axial rotation was minimum. ROM of left lateral bending and right lateral bending was similar. This was consistent with the trend of FE model reported by Lu et al. [14]. FE model used in this study conformed to the physiological characteristics of human body and verified the effectiveness of the model (Fig. 4).

### ROM

**Fig. 5:**
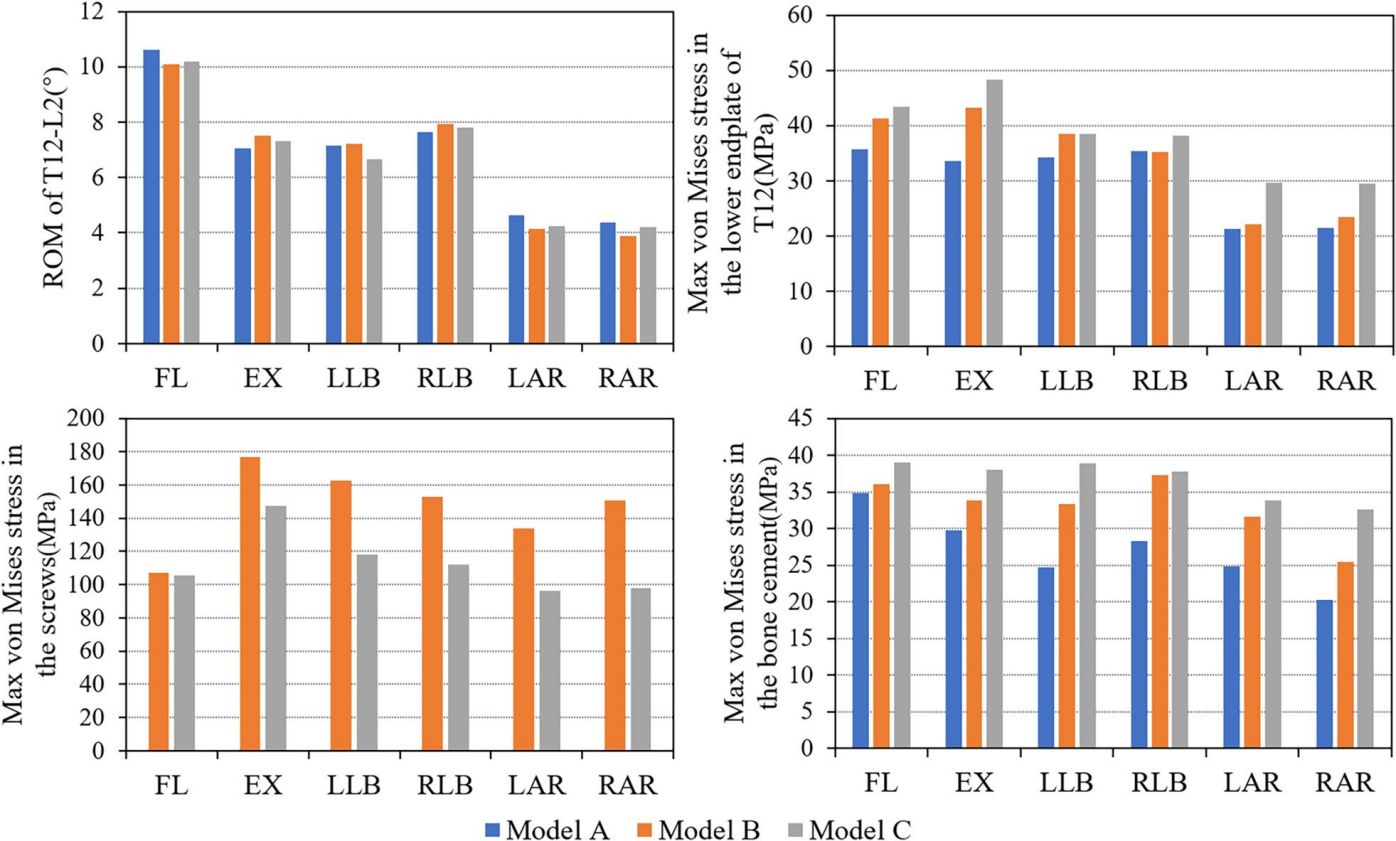
ROM, max von Mises stress in the lower endplate of T12, max von Mises stress in the screws and max von Mises stress in the bone cement in different movement directions of three FE models after operation (FL: flexion, EX: extension, LLB: left lateral bending, RLB: right lateral bending, LAR: left axial rotation, RAR: right axial rotation)

The overall ROM in three postoperative models (T12-L2) was no significant difference (Fig. 5). In all three postoperative models, the maximum ROM was in flexion and the minimum ROM was in rotation. The maximum ROM was 10.62° in flexion (Model A), while the minimum ROM was 3.88° in right axial rotation (Model B).

### Von Mises stress in the lower endplate of T12

**Fig. 6:**
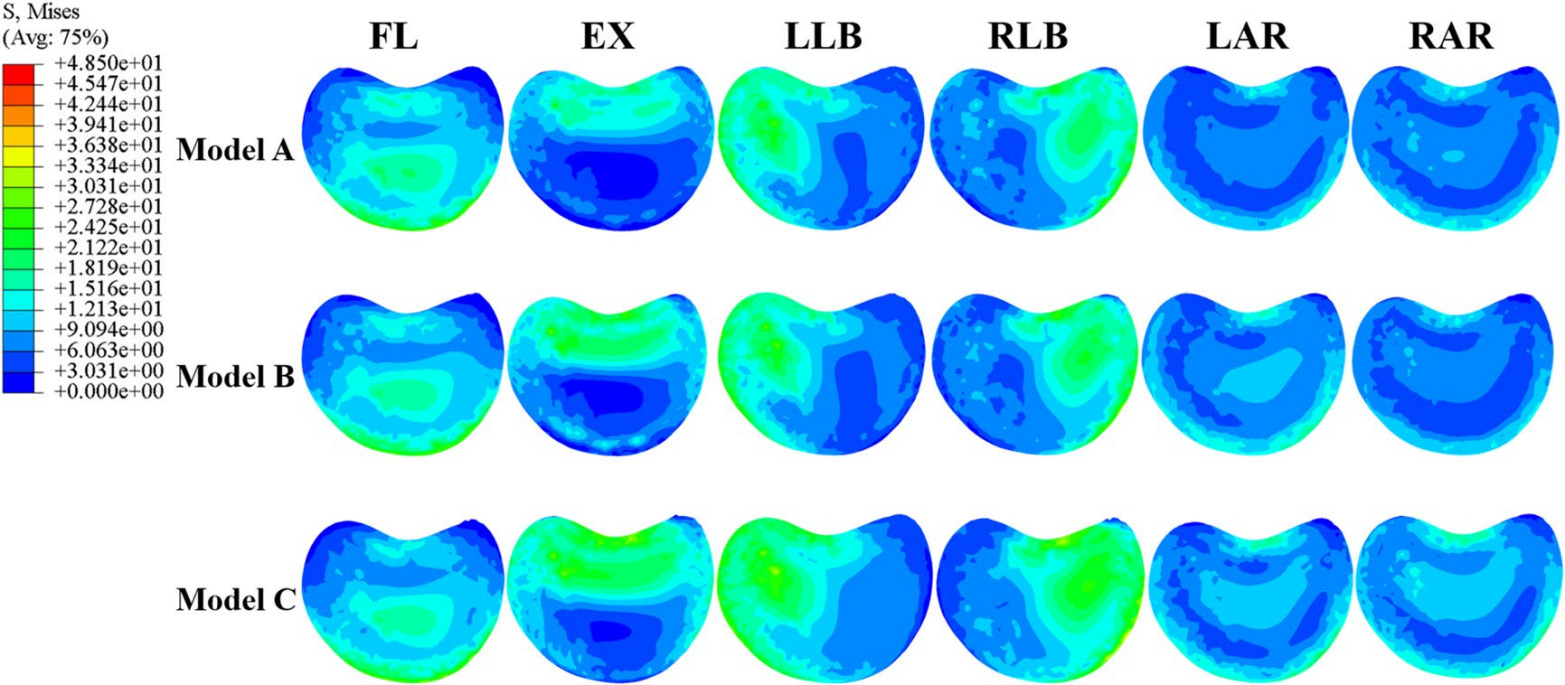
Von Mises stress (MPa) distribution in the lower endplate of T12 of the three models

Compared with pure percutaneous vertebroplasty, maximum von Mises stress in the lower endplate of T12 was increased in the minimally invasive bone cement screw model, and the maximum was found in the bilateral anchorage group (Fig. 5). The stress change was the most obvious in extension. Maximum von Mises stress in the lower endplate of T12 of Model B increased 34.81% and 57.69% in Model C compared with Model A. In all the six directions, maximum von Mises stress in the lower endplate of T12 reached the minimum during rotation in the three models. The maximum stress of endplate was 35.68 MPa (Model A), 43.18 MPa (Model B) and 48.37 (Model C), respectively. In terms of the overall stress distribution of the endplate, there was an obvious stress concentration area in Model C (Fig. 6).

### Von Mises stress in the screws

**Fig. 7:**
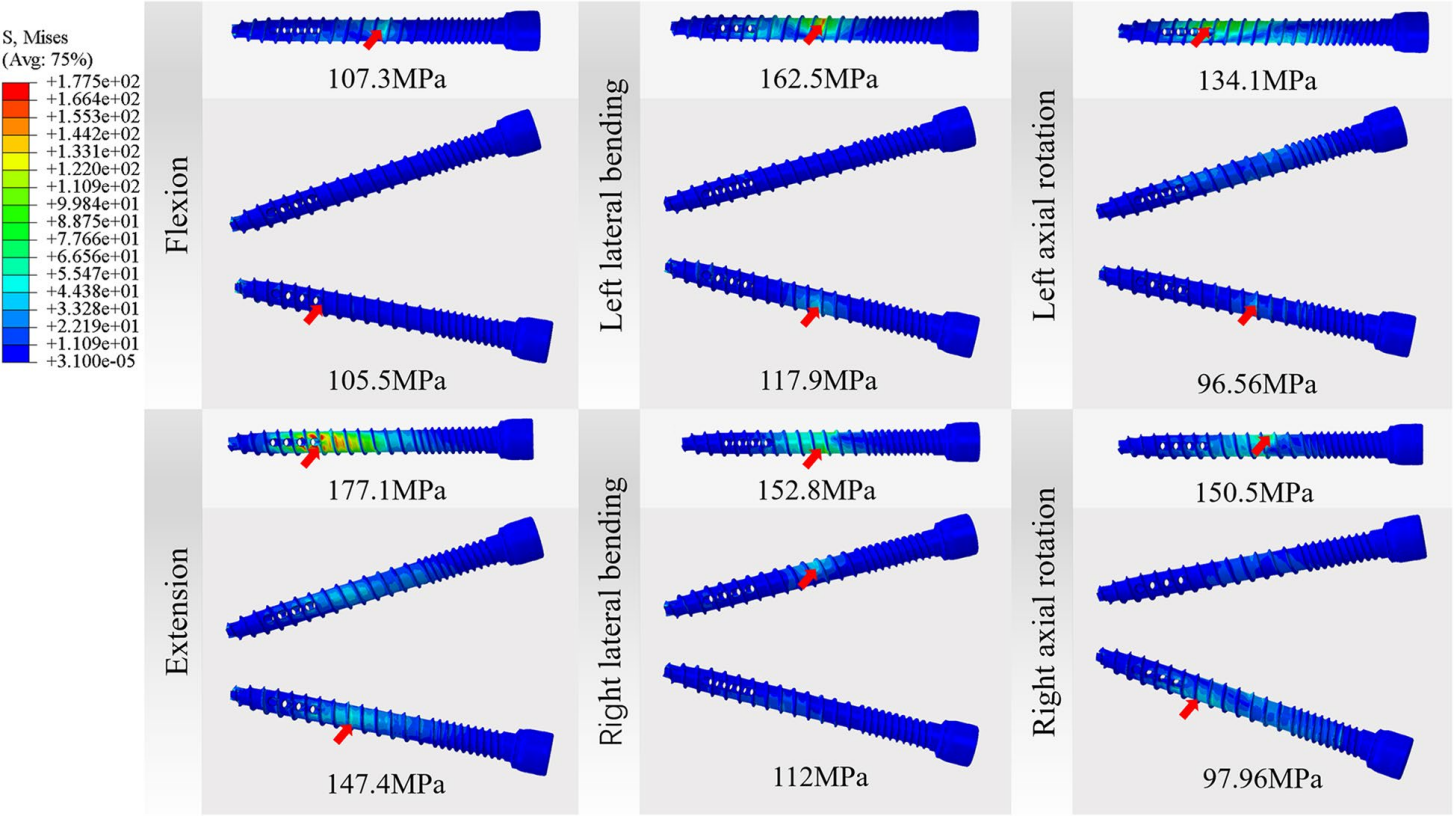
Von Mises stress (MPa) distribution and maximum position of bone cement screws in novel unilateral and bilateral cement screw placement models

In the all movement directions, the maximum Von Mises stress of bone cement screws in Model C was smaller compared with that in Model B (Fig. 5). The movement with the largest proportion of decreasing degree was right axial rotation (34.91%), and the smallest was flexion (1.68%). In both groups of Model B and Model C, the maximum stress of screws appeared in extension. The corresponding Von Mises stress in the screws was 177.1 MPa in Model B and 147.4 MPa in Model C (Fig. 7).

### Von Mises stress in the bone cement

Von Mises stress in the bone cement in the Model B and Model C increased compared with that in Model A, with a greater increase in the Model C (Fig. 5). The bone cement stress in Model B and Model C increased by 28.32% and 43.74% respectively compared with Model A in the left lateral bending. In all six directions, the maximum bone cement stress of Model A was 34.87 MPa in flexion, that of Model B was 34.87 MPa in right lateral bending, and that of Model C was 39.00 MPa in flexion.

### Relative displacement of bone cement

The relative displacement of bone cement in Model B and Model C decreased in all motion directions compared with Model A(Table 2). The relative displacement of bone cement decreased at most 65.83% in flexion and at least 44.39% in extension. With the same movement direction, the relative displacement of bone cement in Model B and Model C had little difference.

**Table 2:** Relative displacement of bone cement during different movements in the three models (mm)

	Model A	Model B	Model C
FL	0.3175	0.1085	0.1075
EX	0.2307	0.1290	0.1283
LLB	0.4992	0.2486	0.2402
RLB	0.4927	0.2573	0.2485
LAR	0.2178	0.0854	0.0795
RAR	0.2283	0.0733	0.0715

## Discussion

KD most often occurs after osteoporosis vertebral compression fracture. Because the vacuum fissure of the vertebral can not heal on its own, the repair mechanism of the diseased vertebral enters a vicious circle, resulting in progressive vertebral collapse, intravertebral pseudoarthrosis, kyphotic deformity and secondary spinal stenosis accompanied by neurological symptoms in severe cases, which is also referred to clinical nonunion of vertebral osteoporotic fracture [20, 21]. The most common affected vertebrae are located at the thoracolumbar junction [22–24]. Some scholars believe that KD is a complication or end-stage manifestation of vertebral compression fracture. Once the disease develops, it would seriously affect the life and quality of life, requiring more aggressive surgical treatment [1].

KD cannot be treated conservatively in most cases due to its non-union characteristics. Although treatment such as percutaneous vertebroplasty (PVP) and percutaneous kyphoplasty (PKP) can stabilize the vertebral body and relieve pain in a short time, the bone cement cannot be firmly anchored on the bone tissue, which is easy to cause the bone cement to loosen during postoperative activities. Mainly because bone cement is mostly filled in the fracture of the vertebral body, the capsule has formed in the fracture, and the bone cement cannot break through the capsule, it cannot be closely bonded with the surrounding bone trabecula. In severe cases, displacement and fracture may occur [25].

In case of fracture and loosening of bone cement, anterior and posterior combined surgery is required. Open reduction titanium mesh implantation with bone grafting and pedicle screw internal fixation are generally selected. This surgical method is characterized by high trauma and high risk, and poses a great threat to the life safety of elderly patients with osteoporosis.

Current operative implants could not simultaneously meet the objectives of minimally invasive treatment and anchoring cement masses to prevent cement displacement, loosening, and fracture. To address these clinical problems, we introduced a new pedicle bone cement screw that could be inserted minimally percutaneous without expanding the incision to expose the vertebral plate, significantly reducing patient injury.

With the application of this pedicle bone cement screw, the puncture needle used in PKP and PVP could be precisely implanted into the diseased vertebra without additional trauma. The front end of the bone cement screw is provided with multiple bone cement exudation holes, which the bone cement can be directly injected into and fused with the injected bone cement by PKP or PVP, so that the screw and the bone cement were firmly anchored.

This novel bone cement screw does not have an excessively long metal tail to irritate the surrounding soft tissue and will not cause pain and discomfort. The diameter of the tail end of the top cap is larger than the diameter of the screw, which prevents the cement mass from moving forward with the screw in the osteoporotic vertebrae. It solved the problem of loosening, displacement and fracture of bone cement mass in KD.

In this study, the biomechanical performance of spinal surgery in daily activities was taken as the starting point, and the influence of this screw on the spinal biomechanical environment after the operation of KD was explored in detail from the internal system. The FE method was used to simulate the postoperative spinal in the movement of flexion, extension, left lateral bending, right lateral bending, left axial rotation and right axial rotation. The results showed that there was no significant difference in the ROM of the three groups of models. Compared with Model A, the relative displacement of bone cement in Model B and Model C was smaller, while maximum equivalent stress of T12 inferior endplate and maximum Von-Mises of bone cement was larger. The stress on the cement screws in Model C was less than that in Model B.

Since the three postoperative models were modified within a single vertebral body, and the operation only involved the L1 vertebral body and did not fix more segments, there was no difference in ROM. A previous research [16] has reported the difference of more than 20% can be called “obvious”. In this study, the relative displacement of bone cement in Model B and C was significantly reduced compared with that in Model A, and the bone cement in both groups had a good fixation effect. The fixation effect of one minimally invasive bone cement screw was basically the same as that of two implants. In addition, in all motion directions of the experiment, the relative displacement of bone cement in the three groups of models reached the maximum during lateral bending, which suggested that Kummell's patients should perform lateral bending more carefully after surgery.

The stress change trend of the lower endplate of T12 and bone cement was similar, increasing gradually from Model A to Model B and then to Model C. The bilateral anchoring group increased the fixation strength of bone cement, and the increasing number of bone cement screws also shared the stress of the fixation system. However, stress of T12 lower endplate and bone cement in bilateral anchoring group was greater. Some studies have shown that load transfer, which could be along the longitudinal axis of the spine to the adjacent vertebral body, is one of the important causal factors affecting the fracture risk of the adjacent vertebrae [26–29]. The anterior column accounts for most of the mechanical conduction of the spine. In this finite element model, to a large extent, the stress of the lower endplate of T12 represents the force exerted by the upper vertebral body on the structure below the lesion level. A smaller value tended to indicate better stability of the L1 vertebra. Figure 5 shows that the bright color areas of the endplate stress cloud images from Model A to Model B and then to Model C gradually increase in all directions of movement, indicating that there were more high stress areas in the endplate of Model C. This means that patients in the bilateral anchoring group are most likely to be unstable in L1 during daily activities. Therefore, the fixed performance benefits brought by bilateral anchoring were accompanied by greater risks.

It can be seen from Fig. 6 that the stress at the side hole of the screw of Model B is significantly higher than that of Model C. It shows that the design of lateral hole plays an important role in the fixation of bone cement screw. Polymethylmethacrylate (PMMA) is a strong but lightweight polymer possessing a compressive strength between 85 and 110 MPa [30]. In the three postoperative models, the maximum stress of bone cement was 38.95 MPa, which was in the safe value. What’s more, yield strength of titanium nail rod was 825-895 MPa, and the destruction intensity of human cortical bone was between 90–200 MPa [5]. In this paper, the maximum stress of bone cement screws in six directions was 177.1 MPa, and the maximum stress of endplate was 43.42 MPa. Although the stress values of both models were within the safe range, this trend might be amplified by the fact. Because the FE study only analyzed the situation at certain conditions and the spine could be subjected to greater forces in everyday life. That is to say, the increase of screw stress in the unilateral anchor model was far from the dangerous value, while the increase of endplate and bone cement screw stress in the bilateral anchor model greatly increases the risk of postoperative instability.

There are still some limitations: first, due to the principle of finite element analysis, the data obtained are more representative of the overall trend. In addition, the ligament and disc structures were reduced to linear elastic properties, but the purpose of this study was to compare the effect of the number of new minimally invasive bone cements applied on the spine, so this simplification had little impact on the comparison results. Finally, the finite element modeling data obtained from individual image data may be biased from individual differences for the whole population. The next research should improve the finite element modeling of complex structures and collect more sample data for analysis.

## Conclusion

The novel minimally invasive bone cement screw can effectively prevent the displacement of bone cement in Kummell therapy. Unilateral anchoring can achieve better fixation effect, and it has less influence on the biomechanical properties of the vertebral body than bilateral anchoring, which provides a reference for the selection of intraoperative instruments.

## Data Availability

The datasets generated and analyzed during the current study are available from the corresponding author on reasonable request.

## Abbreviations

KD: Kummell's disease
PVP: Percutaneous vertebroplasty
PKP: Balloon kyphoplasty
FE: Finite element
3D: 3-Dimensional
ROM: Ranges of motion
ALL: Anterior longitudinal ligament
PLL: Posterior longitudinal ligament
LF: Ligamentum flavum
CL: Capsular ligament
ITL: Intertransverse ligament
ISL: Interspinous ligament
SSL: Supraspinous ligament
FL: Flexion
EX: Extension
LB: Left lateral bending
RB: Right lateral bending
LAR: Left axial rotation
RAR: Right axial rotation
